# Sanxan–Protein Complex Particles for Stabilization of Pickering Emulsions: Improving Emulsification Properties

**DOI:** 10.3390/foods13233854

**Published:** 2024-11-28

**Authors:** Qianqian Wang, Xinyu Wang, Xiaoxi Qi, Libo Liu, Guofang Zhang, Peng Du, Chun Li

**Affiliations:** 1 Key Laboratory of Dairy Science, Ministry of Education, College of Food Science, Northeast Agricultural University, 600 Changjiang Road, Harbin 150030, China; w1872469@163.com (Q.W.); 15846318635@163.com (X.W.); xiaoxi.qi@neau.edu.cn (X.Q.); liboliu@126.com (L.L.); dupeng@neau.edu.cn (P.D.); 2 Heilongjiang Institute of Green Food Science, Northeast Agricultural University, Harbin 150028, China

**Keywords:** sanxan, emulsification, protein, Pickering emulsion

## Abstract

Sanxan (SAN) is a novel microbial polysaccharide that is both safe and edible and represents a promising new source of food resources. It exhibits gelling properties and certain emulsifying properties. To date, there have been few studies published on the enhancement of protein emulsification by sanxan. In this study, three widely used proteins were used: casein (CS), pea protein isolate (PPI), and soy protein isolate (SPI). SAN–protein composite particles were prepared by non-covalent interactions to evaluate the availability of SAN in Pickering emulsions. The effect of SAN on the ability of the complexes to stabilize the emulsion was investigated by measuring and characterizing the physicochemical properties of three SAN–protein complexes. Fourier transform infrared (FTIR) and fluorescence spectroscopy analyses showed that SAN was able to bind to three proteins to form complexes. All three complexes formed by SAN with SPI, PPI and CS had good emulsification properties, with PPI-SAN being the best. Storage results showed better stability of the composite particle-stabilized emulsion. These results indicate that the complexation of SAN with proteins improves the emulsification of proteins and increases the stability of Pickering emulsions. The findings of this study provide valuable information for the utilization of SAN in emulsions.

## 1. Introduction

Pickering emulsions stabilized by solid particles have become popular among researchers due to their excellent stability, completeness, and bioaccessibility [1]. Unlike conventional emulsions, Pickering emulsions can use proteins, polysaccharides, and other naturally sourced substances as emulsifiers. Numerous studies have shown that Pickering emulsions can be used as films [2], fat substitutes [3], nutrient delivery systems, etc. [4], which is strong evidence of their great potential in the food and pharmaceutical fields. Currently, Pickering emulsions’ research focuses on green, regenerative, safe, and sustainable aspects [5]. The development of food-grade granular emulsifiers that can enhance the stability of Pickering emulsions and meet the green health requirements is of great significance to the food industry.

Polysaccharides are naturally occurring carbohydrate polymers that come from a wide range of sources. According to their sources, they can be classified as plant polysaccharides, animal polysaccharides, and microbial polysaccharides. Although they can form a certain spatial structure to stabilize the grease [6], most of them are highly hydrophilic, which is not conducive to further stabilization of the emulsion. Numerous studies have shown that polysaccharides such as starch [7], chitosan [8], pectin [9], cellulose [10], etc., can be chemically modified or complexed with other biopolymers to build stable emulsion systems. In particular, protein–polysaccharide complexes have attracted much attention, and studies have shown that complexes can combine the advantages of proteins and polysaccharides to solve the problem of high stability that is difficult to achieve with single-particle stabilizing emulsions [11,12,13].

Sanxan (SAN) is a novel microbial polysaccharide produced by *Sphingomonas sanxanigenens* NX02, whose monosaccharides are glucose, mannose, rhamnose, and glucuronic acid. The safety and palatability of SAN have been demonstrated and were approved for use as a food additive by the National Health Commission of China in 2020 [14]. SAN exhibits favorable gelling properties and certain emulsifying properties. Liang et al. added SAN to unsalted freeze-cooked pasta and elucidated the mechanism by which SAN improves dough, demonstrating the major role of its thickening properties in this process [15]. Lu et al. used SAN crosslinked with Ca^2+^ to prepare gel beads, which can be used for drug delivery and sustained release [16], and Zhong et al. used SAN as an emulsifier to prepare a novel emulsion gel and demonstrated that the gel could deliver β-carotene [17]. These properties make SAN a promising research prospect in the fields of food and medicine. No studies have been conducted on the complexation of SAN with proteins to improve emulsification properties and stabilize Pickering emulsions.

Casein (CS), soy protein isolate (SPI), and pea protein isolate (PPI) are the most common proteins in Pickering emulsions, with high nutritional value and good functional properties. They possess lipophilic and hydrophilic groups that allow them to adsorb at the oil–water interface and prevent oil droplets from agglomerating [18]. However, emulsions stabilized by single proteins are susceptible to external factors such as temperature, ionic concentration, etc. Therefore, more modification of proteins is required to further improve emulsion stability according to practical applications [19,20,21].

In this study, three proteins, PPI, SPI, and CS, were selected to assess the feasibility of SAN to improve protein emulsification, and complexes of SAN with these three proteins were constructed. The complexes were characterized for structural and physicochemical properties to investigate the changes in the structure of the SAN–protein complexes and the effect on the properties, especially on the improvement of emulsification capacity. The effect of different SAN polysaccharide concentrations on the stability of emulsions was investigated using emulsion stability as an indicator.

## 2. Materials and Methods

### 2.1. Materials

Food grade sanxan (SAN) was purchased from Hebei Xinhe Biochemicals Co., Ltd. (Xingtai, China). Casein (CS) from Beijing Biotopped Technology Co., Ltd. (Beijing, China). Soy protein isolate (SPI) and pea protein isolate (PPI, 80%) were purchased from Shanghai Yuanye Biotechnology Co., Ltd. (Shanghai, China). Soybean oil is purchased from local supermarkets in Harbin, China. Sodium dodecyl sulfate (SDS) is purchased from Beijing Solarbio Science & Technology Co., Ltd. (Beijing, China). The chemicals used in this study are of analytical grade. Deionized water was used for all experiments.

### 2.2. Preparation of Protein–SAN Complexes and Emulsions

Firstly, CS was dispersed in a 0.1 M phosphate-buffered solution, and the pH was adjusted to 7 with a 2 M NaOH solution, stirred for 2 h at 45 °C in a water bath, and hydrated for 12 h at 4 °C to obtain CS solutions. SPI and PPI were dispersed in deionized water, pH adjusted to 7 at room temperature (25 °C) with magnetic stirring for 2 h, and hydrated at 4 °C for 12 h to obtain SPI and PPI solutions. Next, SAN was dispersed in deionized water at room temperature (25 °C) and stirred for 2 h to obtain different concentrations of SAN dispersions. Finally, different concentrations of SAN solutions were mixed with protein solutions in equal volumes at room temperature and stirred for at least 2 h to obtain different protein–SAN complexes. The final concentration of protein in the composite solution was 3% (*w*/*v*), and the final concentration of SAN was 0.1–0.5% (*w*/*v*). The prepared complexes were mixed with soybean oil with an oil phase fraction of 20% (*v*/*v*) and immediately sheared at 12,000 rpm for four minutes using a high-speed shear (T18, IKA, Staufen, Germany) to obtain emulsions stabilized by the different complexes.

### 2.3. Fourier Transform Infrared (FTIR) Spectroscopy

Three complexes of 0.4% SAN with 2% CS, 3% PPI, and 3% SPI were prepared. The sample of the complex was freeze-dried and ground into powder. The samples were accurately weighed at 1:150 and mixed with potassium bromide, then ground thoroughly and pressed into thin tablets using a tablet press. Measurements were carried out with a Nicolet iS50 FTIR spectrometer (Thermo Fisher, Waltham, MA, USA) at an ambient temperature of 25 °C with a resolution of 4 cm^−1^ and a scanning spectrum of 4000–400 cm^−1^ with 32 scans [22].

### 2.4. Endogenous Fluorescence Spectroscopy

The fluorescence intensity of the proteins, SAN, and their complexes was measured using an RF6000 fluorescence spectrophotometer (Shimadzu, Kyoto, Japan). The equipment measurement conditions were an excitation wavelength of 280 nm, an excitation slit width of 5 nm, an emission slit width of 5 nm, and a scanning range of 300–500 nm [23].

### 2.5. Determination of Particle Size and Zeta Potential

Dilute the sample with deionized water before measurement. The average particle size and zeta potential of the dilution samples were measured using a nanoparticle size and zeta potential analyzer (Zetasizer Nano, Malvern, Malvern, UK) at room temperature, three times for each sample.

### 2.6. Emulsion Activity and Emulsion Stability

The method for the determination of emulsification activity (EAI) and emulsion stability (ESI) was based on the method by Wang et al. [24] with some modifications. The emulsion was prepared according to Section 2.2. Moreover, 50 μL of freshly prepared emulsion (pH = 7) was pipetted at a fixed position in a beaker, the emulsion was diluted 100-fold with 0.1% (*w*/*v*) SDS solution, and the absorbance value was measured as A_0_ at a wavelength of 500 nm. The absorbance value of the emulsion measured by repeating the above steps after 10 min of standing was *A*_10_. The absorbance values of SDS solution (0.1%, *w*/*v*) were used as a blank set. The formulas for *EAI* and *ESI* were as follows:

(1) $$EAI/(m^{2}/g)=\frac{2\times 2.303\times A_{0}\times N}{C\times (1-\emptyset )\times 10^{4}}$$

(2) $$ESI\left(\%\right)=\frac{A_{10}}{A_{0}}\times 100$$

where *N* is the number of dilutions (100); ∅ is the volume fraction of the oil phase in the system (0.2); and *C* is the emulsifier concentration (g/mL).

### 2.7. Contact Angle Measurement

Referring to the method of Wang et al. [25], dried samples of proteins, SAN, and their complexes were compressed into thin slices of about 1–2 mm thickness and 10.0 mm diameter at room temperature. Precisely 5 μL of pure water was dropped on the surface of the sample, the contact angle of the sample was measured, and the shape of the droplet was recorded using a Theta Optical Contact Angle Measuring Instrument (Biolin, Gothenburg, Sweden). Each sample was measured three times.

### 2.8. Scanning Electron Microscopy (SEM)

The protein, SAN, and complex samples were lyophilized and sprayed with gold, and the microstructures of the samples were observed with an S-3400N tungsten filament scanning electron microscope (Hitachi, Tokyo, Japan) at an accelerating voltage of 5 kV.

### 2.9. Emulsion Stability

#### 2.9.1. Centrifugal Stability

An aliquot of 5 mL of freshly prepared emulsion was taken in a 10 mL centrifuge tube and centrifuged at 5000 rpm for 5 min. The total height of the emulsion and the height of the supernatant before and after centrifugation were determined. All samples were repeated three times. The calculation formula for *CI* was as follows [26]:

(3) $$CI\left(\%\right)=\frac{H_{S}}{H_{T}}\times 100$$

where *H_S_* is the height of the lower scavenge and *H_T_* is the total height of the emulsion.

#### 2.9.2. Storage Stability

The freshly prepared emulsions stabilized with different complexes were placed in clear glass vials and stored at room temperature (25 °C) and 37 °C, respectively, protected from light. Changes in appearance were observed at intervals of 7, 14, 21, and 28 d and photographed and recorded in front of a black background plate.

#### 2.9.3. Thermal Stability

Under the experimental conditions, pasteurization was performed at 63 °C for 30 min [27]. In order to evaluate the thermal stability of the emulsions at 63 °C. Glass vials containing freshly prepared emulsions were placed in a water bath at 63 °C for 1 h. The vials were taken out after 30 min and 60 min to observe the changes in appearance and photographed for recording.

### 2.10. Statistical Analysis

All experiments were repeated three times, and the results are expressed as mean ± standard deviation. One-way analysis of variance (ANOVA) and Duncan’s multiple tests were performed using SPSS 25.0 (IBM, Chicago, IL, USA) software. *p* < 0.05 indicates a significant difference. Plotting was performed using Origin 2022 (OriginLab, Northampton, MA, USA).

## 3. Results and Discussion

### 3.1. Formation of Three Protein–SAN Complexes

#### 3.1.1. FTIR Spectroscopy Analysis

**Figure 1:**
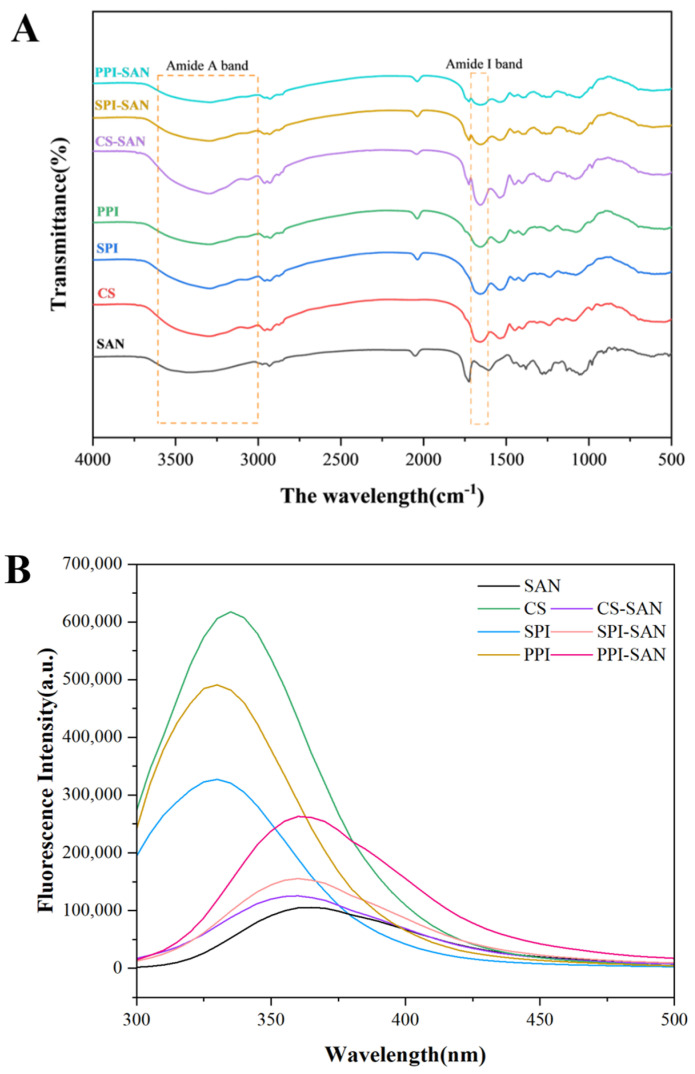

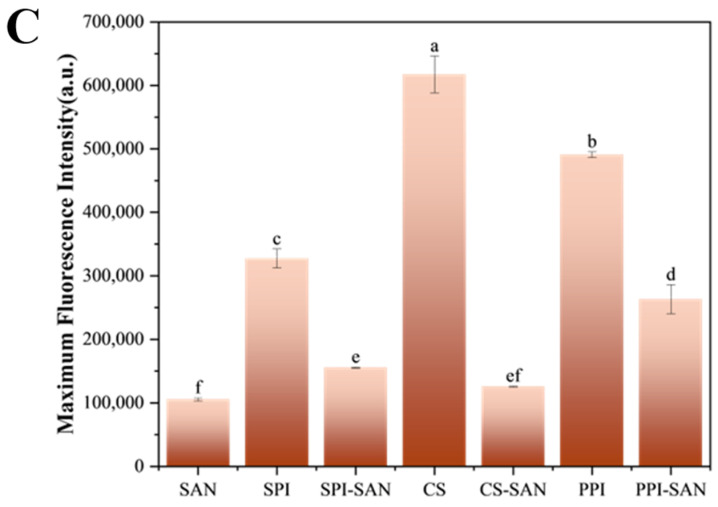
Fourier infrared spectra (**A**), fluorescence spectra (**B**), and maximum fluorescence intensity (**C**) of three proteins (CS, SPI, and PPI), SAN, and protein–SAN complexes (CS-SAN, SPI-SAN, and PPI-SAN). Different small superscript letters indicate the presence of statistically significant differences (*p* < 0.05).

The FTIR spectra of the three proteins, SAN, and three protein–SAN complexes are shown in Figure 1A. By analyzing the characteristic absorption bands in the spectra, it is possible to reflect the changes in the structure of the proteins and their interactions after the addition of SAN [28]. The most important characteristic absorption bands of proteins are the amide A band (3600–3000 cm^−1^), amide B band (3000–2800 cm^−1^), amide I band (1700–1600 cm^−1^), and so on, where amide A carries reactive O-H stretching and N-H stretching [29]. The absorption peak of the amide A band of the three proteins was in the wavelength range of 3283.22–3297.68 cm^−1^. Compared with PPI (3285.63 cm^−1^), SPI (3283.22 cm^−1^), and CS (3297.68 cm^−1^), the absorption peak wavelengths of the amide A bands of the complexes PPI-SAN (3309.25 cm^−1^), SPI-SAN (3288.52 cm^−1^), and CS-SAN (3305.88 cm^−1^) showed a slight redshift. This is due to the increased hydroxyl content of the reaction system of SAN with the three proteins, which in turn creates hydrogen bonding [30]. Due to the deformation vibration of O-H [31], SAN shows a strong absorption peak at 1723.57 cm^−1^. This peak is also present for the three protein–SAN complexes, which can indicate that binding of SAN to the three proteins occurs. Except for the characteristic peak of SAN, the three complexes CS-SAN, PPI-SAN, and SPI-SAN did not show any new peaks compared with the three proteins, CS, PPI, and SPI, which indicates that no new substances were produced. In addition, the characteristic peak of the amide I band of the complex was slightly shifted towards the long wave (1658.96–1660.41 cm^−1^) compared to the characteristic peak of the amide I band of the three proteins (1654.14–1655.55 cm^−1^). This may be due to changes in the secondary structure of the proteins and possible electrostatic interactions between SAN and the three proteins [32].

#### 3.1.2. Fluorescence Spectroscopy Analysis

Changes in fluorescence spectra are mainly caused by changes in tryptophan residues and can be used to respond to changes in protein tertiary conformation and complex formation [33]. As shown in Figure 1B, CS, SPI, and PPI all have strong maximum emission peaks. The maximum fluorescence intensity of all three proteins was significantly reduced by the addition of SAN (Figure 1C). The maximum fluorescence intensity of CS-SAN (125,589.90) was 80% lower than that of CS (617,351.41); SPI-SAN (155,373.11) was 53% lower than that of SPI (327,294.90); and PPI-SAN (263,101.88) was 46% lower than that of PPI (490,865.15). The occurrence of fluorescence quenching indicates that SAN interacts with the three proteins and leads to a change in protein conformation [34], which is consistent with the results of Fourier transform infrared (FTIR) spectrograms. In addition, the maximum absorption wavelength of the complex was red-shifted. This may be due to the fact that SAN interacts with the protein, exposing more tryptophan residues on the surface of the protein [35], or it may be due to the fact that their interaction causes the protein to undergo rearrangement [36].

### 3.2. Zeta Potential Analysis of Complexes

**Figure 2:**
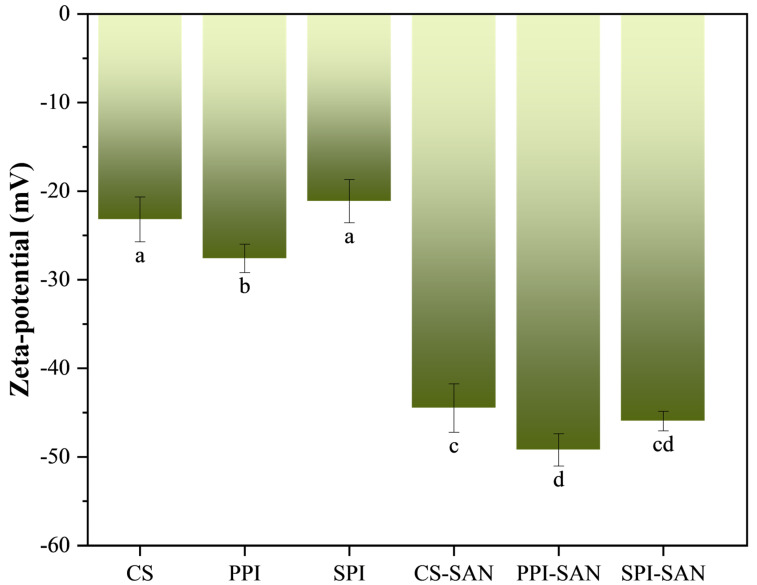
Zeta potential of three proteins (CS, SPI, and PPI) and three complexes (CS-SAN, SPI-SAN, and PPI-SAN). Different small superscript letters indicate the presence of statistically significant differences (*p* < 0.05).

Zeta potential is an important indicator of the emulsification properties of proteins. The higher the absolute value of the potential, the stronger the electrostatic repulsion of the particles, preventing the occurrence of molecular aggregation and improving emulsion stability [37]. The zeta-potential maps of the three proteins (i.e., CS, SPI, and PPI) and the three complexes (i.e., CS-SAN, SPI-SAN, and PPI-SAN) are shown in Figure 2. The potential values for the three proteins were CS (−23.2 mV), SPI (−21.13 mV), and PPI (−27.6 mV). The potential values of the complexes were CS-SAN (−44.49 mV), SPI-SAN (−45.97 mV), and PPI-SAN (−49.22 mV). The results showed that the addition of SAN resulted in a significant increase in the absolute value of zeta potential for all three complexes. CS, PPI, and SPI are negatively charged, and the anionic polysaccharide SAN undergoes electrostatic repulsion with the three proteins. This prevents the aggregation of protein particles and facilitates the stabilization of the system [38]. Hydrogen bonds are formed between SAN and proteins, which can be seen by the FTIR results. The particle size of the complex is larger than that of the protein, which indicates that the electrostatic repulsion between the protein and the SAN is much weaker than the hydrogen bonding and hydrophobic forces between them. Therefore, the absolute value of the potential and the particle size of the complex are both increased compared to the protein. Among them, the lowest potential value of the PPI-SAN complex was −49.22 mV, which indicated that SAN had a greater influence on the alteration of the protein structure of PPI, which agreed with the results of FTIR. Emulsions prepared from complexes possessing high absolute values of potential have higher stability [39], thus PPI-SAN complex emulsions will have better stability.

### 3.3. Contact Angle of the Complexes

**Figure 3:**
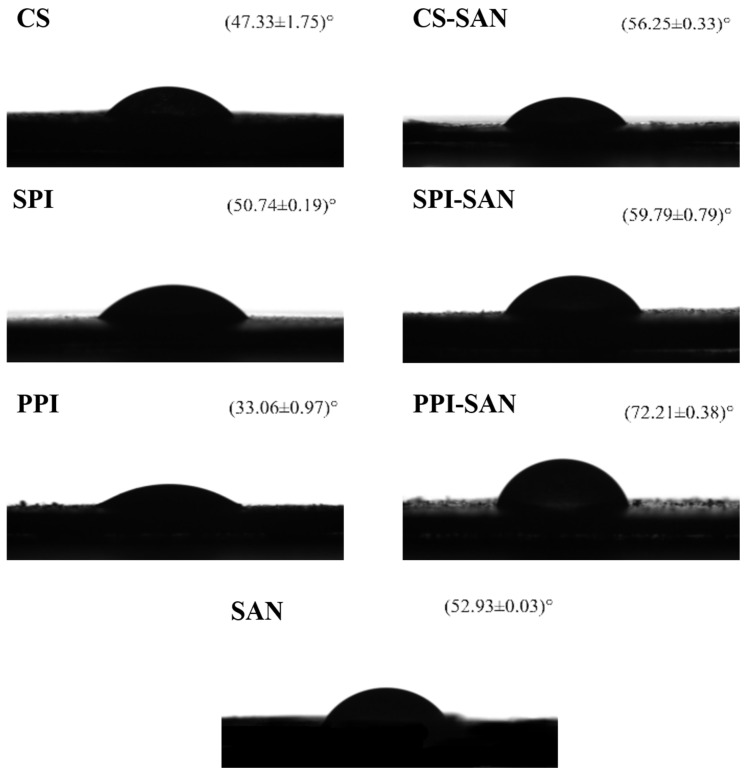
Contact angle images of three proteins, SAN, and protein–SAN complexes.

The size of the contact angle is an important indicator for assessing the ability of particles to stabilize emulsions. The closer the contact angle θ is to 90°, the more balanced the hydrophilic and lipophilic properties of the particles are and the more stable the Pickering emulsion formed [40]. As shown in Figure 3, the contact angles of single particles were CS (47.33 ± 1.75)°, SPI (50.74 ± 0.19)°, PPI (33.06 ± 0.97)°, and SAN (52.93 ± 0.03)°. The complex contact angles were CS-SAN (56.25 ± 0.33)°, SPI-SAN (59.79 ± 0.79)°, and PPI-SAN (72.21 ± 0.38)°. The contact angle of SAN with the protein complexes is greater than that of a single particle, and the surface hydrophobicity of all three proteins is increased. This may be attributed to SAN, protein–protein interactions exposing the non-polar amino acid groups of the protein [41]. Among them, the PPI-SAN contact angle of 72.21° is closest to 90°. In contrast, PPI-SAN has a greater ability to stabilize Pickering emulsions. This result is consistent with the zeta potential results. In addition, the contact angles of all three proteins are smaller than the contact angle of SAN. This is because SAN is amphiphilic, whose emulsifying property is higher than that of proteins [17].

### 3.4. EAI and ESI of the Complexes

**Figure 4:**
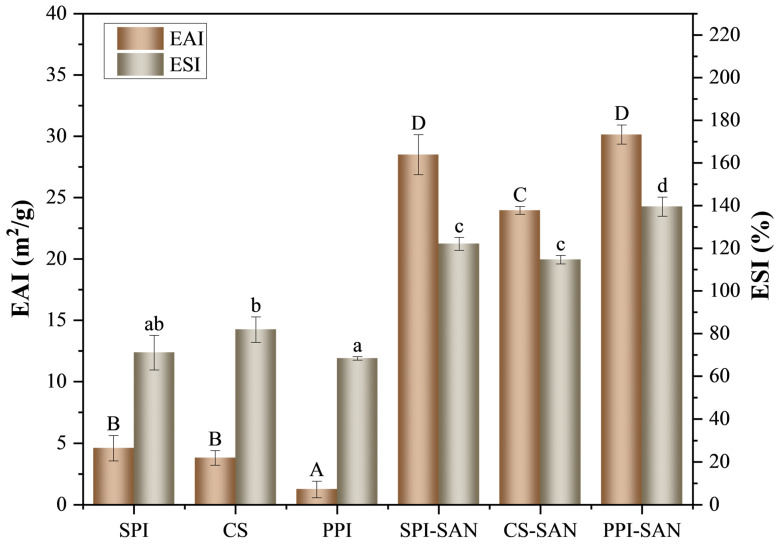
Emulsion activity and emulsion stability of three proteins and three complexes. Different small superscript letters indicate statistically significant differences in ESI for different proteins and complexes (*p* < 0.05). Different capital letters indicate statistically significant differences in EAI for different proteins and complexes (*p* < 0.05).

The EAI and ESI of the complexes are shown in Figure 4. The EAI indices of the emulsions were significantly increased by the addition of SAN as compared to the proteins alone: 23.95 m^2^/g for CS-SAN, 29.16 m^2^/g for SPI-SAN, and 29.80 m^2^/g for PPI-SAN. This greatly improved the emulsifying activity of the proteins, in agreement with previous analyses by fluorescence spectroscopy. The addition of SAN exposes the hydrophobic groups of proteins and enhances the ability to stabilize oil droplets. Because SAN is an amphiphilic polysaccharide, it also favors emulsification activity [38]. In addition, the incorporation of SAN also improved the emulsion stability, where the ESI of PPI-SAN was 117.78%. This may be attributed to the higher absolute value of zeta potential of the complexes [42], stronger electrostatic repulsion, and spatial site resistance improving the stability of the emulsion.

### 3.5. Scanning Electron Microscopy (SEM) Analysis

**Figure 5:**
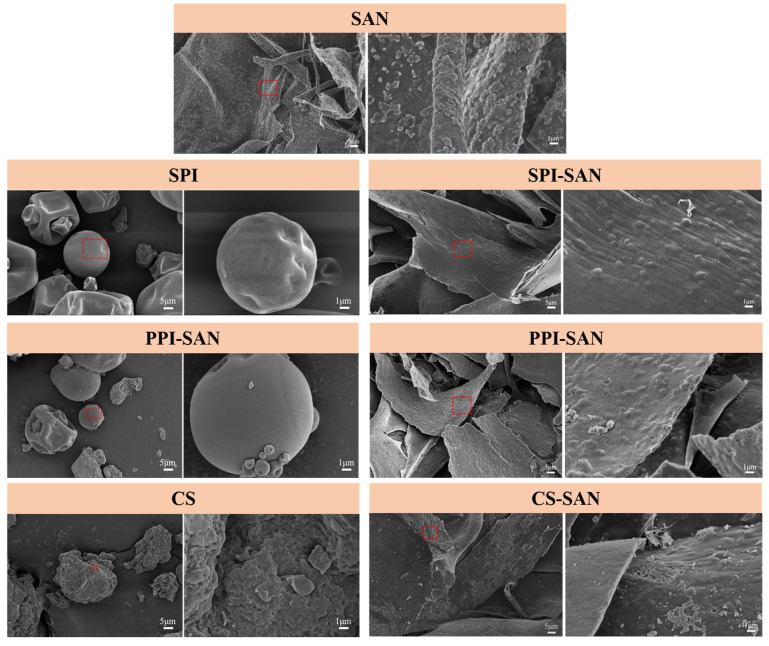
SEM images of SAN, proteins, and protein–SAN complexes. The red dotted box area is the area of the multiples 5K image.

The microstructure of SAN and its complexes with the three proteins is shown in Figure 5. SAN is loosely structured in the form of flakes and has a rough surface. Rough surfaces are probably residual *S. sanxanigenens* cells [14]. SPI and PPI exhibit the appearance of spherical particles [43,44], and CS has an almost spherical appearance [45]. After forming a complex with SAN, the protein particles are embedded into the structure of SAN, giving the surface a number of spherical projections. Moreover, the structure of the complex presents large flakes, which are relatively more homogeneous, which facilitates the stabilization of the Pickering emulsion [46].

### 3.6. Stability of Protein–SAN Pickering Emulsions

#### 3.6.1. Particle Size and Zeta Potential of the Complexes

**Figure 6:**
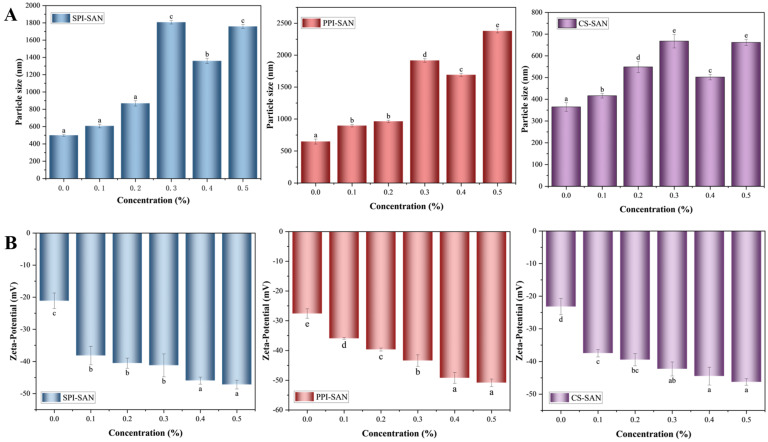
Particle size (**A**) and Zeta potential (**B**) of proteins and different concentrations of SAN complexes. Different small superscript letters indicate the presence of statistically significant differences (*p* < 0.05).

The complex particle size is an important indicator for evaluating its ability to stabilize emulsions. The smaller particle size increases the specific surface area of the stabilized emulsion and favors the stability of Pickering emulsions [47]. The effect of SAN concentration on the particle size of the complexes is shown in Figure 6A. When no polysaccharide was added, the particle size was 498 nm for SPI, 647 nm for PPI, and 364 nm for CS. The particle size of all three groups of protein–SAN complexes tended to increase as the concentration of SAN increased. Hydrogen bonding between the carboxyl group of SAN and the hydrogen acceptor of the protein increases the particle size of the complex [38,48], and the Fourier infrared transformation also confirms the presence of hydrogen bonding in the complex. The increasing concentration of SAN leads to more and more hydroxyl groups in the system, which leads to an increase in the particle sizes of the complexes [49]. When the concentration of SAN was 0.4%, the particle sizes of SPI-SAN (1359 nm), PPI-SAN (1692 nm), and CS-SAN (502 nm) complexes were all reduced (*p* < 0.05), at which time the protein and SAN formed a tightly structured complex [50], and the emulsion was more stable. The particle sizes of the complexes increased again when the concentration of SAN was further increased, which may be due to the excess of SAN in the system, whose own high viscosity increases the particle sizes [51]. As a result, the complexes formed by 0.4% SAN with proteins have a smaller particle size and are more conducive to the formation of stable emulsions.

The zeta potential of the particles can reflect the stability of the solution system. The absolute values of the potentials of all complexes gradually increased with increasing SAN concentration (Figure 6B). This is because the addition of anionic polysaccharide SAN increases the amount of negative charge on the surface of the complex, and the electrostatic repulsion between the complexes increases in favor of the stability of the system [52]. When the concentration of SAN was 0.4%, the potentials of the complexes reached SPI-SAN (−45.97 mV), PPI-SAN (−49.22 mV), and CS-SAN (−44.49 mV), respectively. With a further increase in SAN concentration, the increment in the absolute value of complex potential decreases gradually. This may be due to the high viscosity of the SAN solution, which reduces the mobility of the particles and attenuates the potential difference [53].

#### 3.6.2. Centrifugal Stability of Emulsions

**Figure 7:**
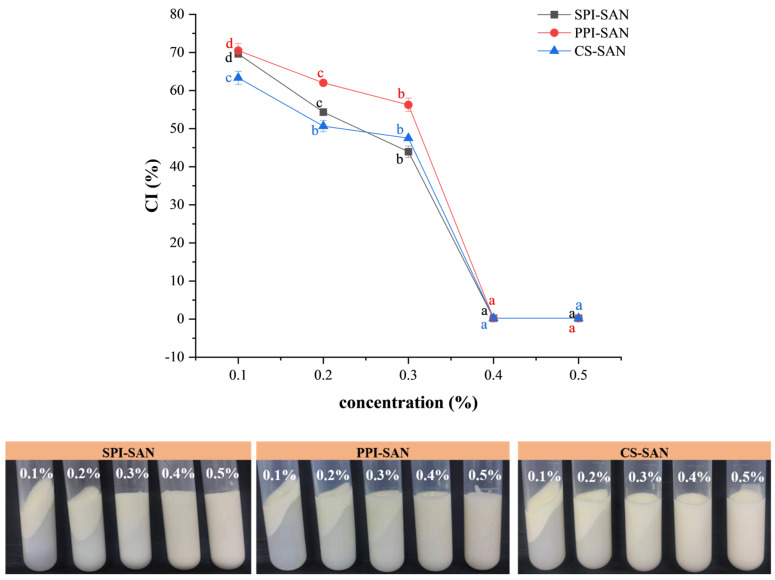
Creaming indices (CI) and appearance of emulsions with different SAN concentrations. Different small superscript letters indicate the presence of statistically significant differences (*p* < 0.05).

Centrifugal stability evaluates the stability of emulsions under mechanical forces. The effect of SAN concentration on the centrifugal stability of the emulsion is shown in Figure 7. The emulsification indices of SPI-SAN, PPI-SAN, and CS-SAN emulsions decreased significantly with increasing SAN concentration. The concentration of SAN affects the centrifugal stability of the emulsion. Emulsions with SAN concentrations less than 0.4% underwent varying degrees of phase separation after centrifugeation, and the smaller the SAN concentration, the higher the aqueous layer after centrifugation and the more unstable the emulsion. When the concentration of SAN is 0.4%, the emulsion does not precipitate after centrifugation. This phenomenon can be attributed to the formation of a dense interfacial layer of complexes within the system, which effectively inhibits the aggregation and coagulation of oil droplets [54], resulting in better centrifugal stability of the emulsion. When the concentration of SAN is 0.4%, the emulsion is basically free of delamination after centrifugation, in which the emulsion has better centrifugal stability. Similar results were obtained in a study by Z. Wang et al. [55], where emulsions stabilized by protein–polysaccharide particles were more stable than single-protein emulsions, and emulsions with high polysaccharide additions had better centrifugal stability. In summary, 0.4% SAN complexed with the three proteins is more beneficial for emulsion stabilization.

#### 3.6.3. Storage Stability of Emulsions

**Figure 8:**
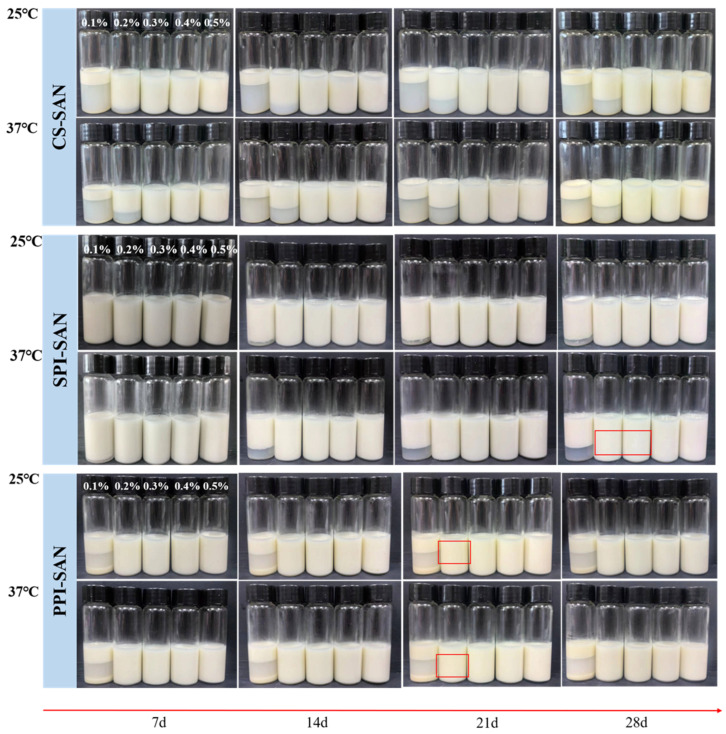
Appearance of emulsions with different SAN concentrations stored at 25 °C and 37 °C for 28 d. The areas in red boxes are areas of light precipitation.

The freshly prepared emulsions were stored at 25 °C and 37 °C for 28 d to observe their stability. The 28 d storage appearance of emulsions with different SAN concentrations is shown in Figure 8. When SAN ≤ 0.2%, CS-SAN emulsion has undergone obvious delamination when stored at 25 °C for 7 d, and the appearance of other concentration emulsions did not change. At an SAN concentration of 0.1%, SPI-SAN emulsion exhibited delamination when stored at 37 °C for 7 d. Slight precipitation was observed on the surface of SPI-SAN emulsion with a SAN concentration of 0.3% when stored at 37 °C for 28 d. In the case of PPI-SAN emulsion, 0.1% SAN was not sufficient to obtain a stable emulsion, and delamination was evident at 25 °C for 7 d. When the concentration reached 0.2%, the emulsion showed surface precipitation at 28 d. The concentration of SAN affects the storage stability of the emulsion; under certain conditions, the greater the concentration of SAN, the more stable the emulsion. This phenomenon may be attributed to the high viscosity of SAN, which restricts the movement of oil droplets and thereby enhances the stability of the emulsion [56]. From the appearance graph, it can be seen that the addition of SAN can improve the storage stability of the emulsion, and the reasonable selection of SAN concentration is more conducive to the generation of stable emulsions. The important role of solid particle concentration on emulsion stability was also demonstrated by C. Yan et al. [57].

#### 3.6.4. Pasteurization Stability of Emulsions

**Figure 9:**
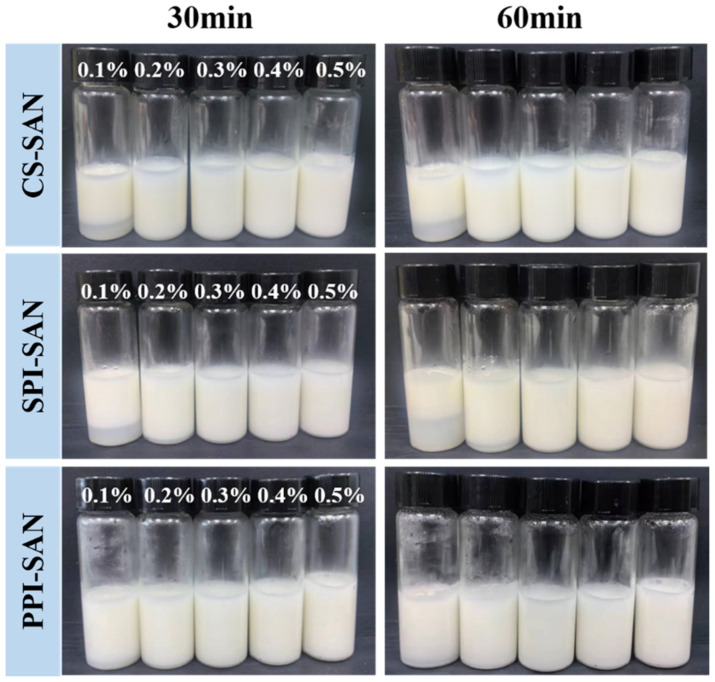
Appearance of emulsions with different SAN concentrations treated at 63 °C for 30 min and 60 min.

Most of the products in the food industry are subjected to pasteurization. The pasteurization step was simulated by heating in a water bath, and the stability of the emulsion at 63 °C was evaluated by visual observation. For all three groups of emulsions, SAN concentrations higher than 0.2% are more resistant to temperature. However, emulsions stabilized at SAN of 0.1% were difficult to resist pasteurization treatment (Figure 9). In summary, the emulsion stabilized by protein complexed with 0.4% SAN was able to maintain excellent stability even at 63 °C for 1 h. Geng et al. studied soy isolate protein–pectin complexes and soy isolate protein–sodium alginate complexes and confirmed that the addition of polysaccharides improved the thermal stability of the emulsions [58].

## 4. Conclusions

In this study, protein emulsification was improved based on the non-covalent binding of the microbial polysaccharide SAN to proteins and applied to Pickering emulsions. The emulsification of three proteins (CS, PPI, and SPI) was improved after SAN modification; among them, PPI demonstrated the most substantial enhancement in emulsification. The findings of this study demonstrated that the Pickering emulsions prepared from SAN–protein particles exhibited excellent stability, with SAN playing a pivotal role in this process. In the future, we intend to apply the Pickering emulsion prepared by SAN as a delivery bioactive ingredient system to provide more possibilities for its application research.

## Data Availability

The original contributions presented in the study are included in the article, further inquiries can be directed to the corresponding author.
